# Impact of Short-Stay Urethroplasty on Health-Related Quality of Life and Patient's Perception of Timing of Discharge

**DOI:** 10.1155/2015/806357

**Published:** 2015-10-01

**Authors:** Henry Okafor, Dmitriy Nikolavsky

**Affiliations:** Department of Urology, Upstate Medical University, 750 East Adams Street, Syracuse, NY 13210, USA

## Abstract

*Objective.* To evaluate health-related quality of life in patients after a short-stay or outpatient urethroplasty. *Methods.* Over a 2-year period a validated health-related quality-of-life questionnaire, EuroQol (EQ-5D), was administered to all patients after urethroplasty. Postoperatively patients were offered to be sent home immediately or to stay overnight. Within 24 hours after discharge they were assessed for mobility, self-care, usual activities, pain or discomfort, and anxiety and depression. An additional question assessing timing of discharge was added to the survey. Clinical and operative characteristics were examined. *Results.* Forty-eight patients after anterior urethroplasty completed the survey. Mean age and mean stricture length were 51.6 years (21–78) and 60 mm (5–200 mm), respectively. Most etiologies were idiopathic (50% *n* = 24), trauma (19%, *n* = 9), and iatrogenic (19%, *n* = 9). Forty-one patients (85%) stayed overnight, while 7 patients (15%) chose to be discharged the same day. Overall, ninety-six percent were discharged within 23 hours of surgery. In the short-stay and the outpatient cohorts, 90% and 86%, respectively, felt they were discharged on time. No patient reported a severe problem with postoperative pain or mobility. *Conclusions.* The majority of patients discharged soon after their procedure felt that discharge timing was appropriate and their health-related quality of life was only minimally affected.

## 1. Introduction

Urethroplasty is recognized as the gold standard treatment of anterior urethral stricture disease, given the reasonably high long-term success rates and acceptable morbidity [1–3]. While, traditionally, urethroplasty was followed by inpatient hospital stay, there has been an increasing trend for urological procedures to be performed on an outpatient basis [4], a pattern reflected in urologic reconstruction as well [5, 6]. While numerous studies have been published reporting the clinical outcomes of urethroplasty, patient perception, satisfaction, and subjective outcomes are not well studied. There is also a paucity of data examining patient perception of early return home from the hospital. The purpose of this study was to examine the patient's perception of appropriateness of timing of discharge and to evaluate immediate health-related quality of life immediately after discharge.

## 2. Methods

With institutional board review approval, electronic charts of 80 consecutive patients who underwent anterior urethral reconstruction at our institution from August 2012 to May 2014 were analyzed. Patients under 18 years of age, those with documented intellectual disability, incarcerated patients, and transgender patients were excluded, as were patients with planned multistage procedures.

All patients underwent preoperative evaluation with retrograde urethrogram and/or voiding cystourethrogram, uroflowmetry, and AUA symptom scores. All patients were counseled at the time of the preoperative evaluation of possible immediate postoperative discharge or overnight stay based on their postoperative condition and desire. Patients were assured that from our previous experience most prior patients safely returned home either immediately following urethroplasty or after an overnight stay providing pain is controlled and there are no other health concerns. Patients were educated on proper use of all postoperative medications and care for Foley catheter. Each patient was given contact information for the clinic and additionally a mobile phone number of the surgeon and were encouraged to call with additional questions or concerns before or after the surgery. The same points were reiterated immediately before the surgery in the Preoperative Unit.

The type of urethroplasty performed was dependent on stricture length, location, and etiology as well as surgeon preference. For substitution or augmentation urethroplasties, only buccal mucosal grafts (BMG) were used. The BMG was harvested as described by Morey and McAninch [7]; however, the harvest site was left open after harvest. The midline perineal incisions were closed in layers and no wound drains were used. A urethral catheter was left in place in all patients. When present, a suprapubic catheter remained capped on discharge.

In the postanesthesia recovery area all patients were assessed and given a choice of immediate discharge or an overnight hospital stay. Patients who elected to return home on the day of surgery were placed in the “outpatient cohort” while those who stayed overnight were a “short-stay” group. Discharge criteria in both groups included hemodynamic stability, adequate pain control with oral analgesics, and sufficient mobility to ambulate without difficulty. Patients were routinely sent home with prescriptions for nonsteroidal anti-inflammatory agents, oral narcotic medications for breakthrough pain, anticholinergics, stool softeners, and anesthetic/antiseptic mouthwash.

Within 24 hours of discharge, a routine postoperative check was conducted over the phone by a nurse or nonmedical administrative assistant. The assessment included questions from the EuroQol (EQ-5D), a validated health-related quality of life (QOL) questionnaire [8, 9]. The questions are designed to assess mobility, self-care, usual activities, pain or discomfort, and anxiety/depression. The choices were scored from 1 to 3 as having “no problems,” “moderate,” or “severe problems,” respectively. An additional question assessing perception of the timing of discharge as “right on time,” “too soon,” or “too late” was added to the interview.

We also reviewed the charts for hospital readmissions, emergency room visits, and unplanned clinic visits to capture any additional potential burden on patients or the healthcare system due to early postoperative discharge.

## 3. Results and Discussion

A total of 48 patients who underwent anterior urethroplasty between August 2012 and May 2014 were included. Mean age of the group was 51.6 years (21–78). Mean stricture length was 59.7 mm (5–200 mm). Preoperative patient characteristics and stricture etiology are shown in Table 1. The most common type of repair was a single stage, one sided dissection, dorsal onlay buccal urethroplasty in 13 (27%) patients as described by Kulkarni [10], followed by excision and primary anastomosis in 11 (23%) and augmented anastomotic urethroplasty in 10 (21%) patients (Table 2). Overall, 37 of the 48 patients (77%) had buccal mucosa harvest for augmentation or substitution urethroplasty of which 8 required bilateral buccal mucosa harvest.

Forty-one patients made a postoperative decision to stay overnight, while seven elected to return home the same day. All except two patients (96%) were discharged within 23 hours of surgery.

**Figure 1:**
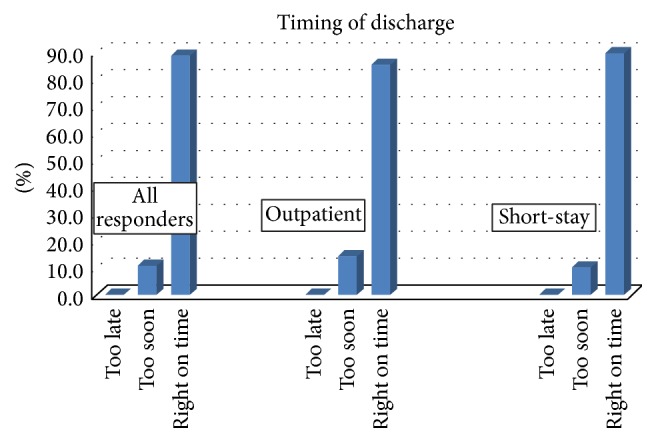
Timing of discharge.

Forty-six out of 48 patients (96%) responded to the EuroQuol-5 questionnaire as well as the question on timing of discharge within 24 hours of discharge. Overall, 89.1 % of all patients felt they were discharged on time (Figure 1).

**Figure 2:**
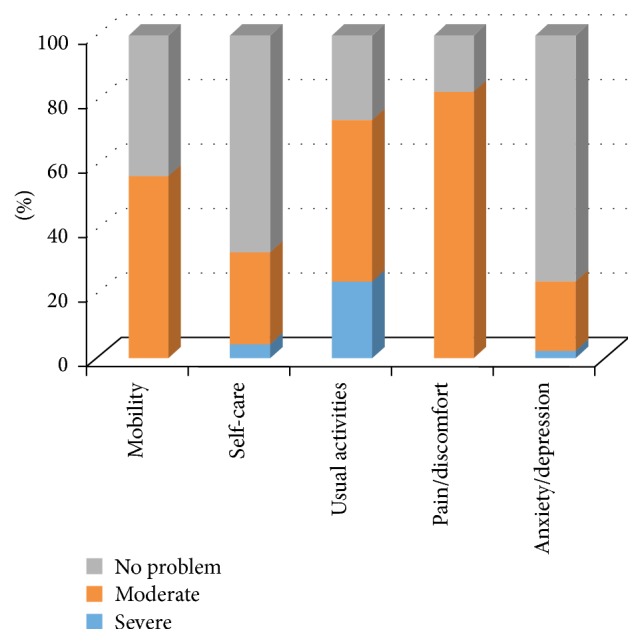
EQ-5 patient responses (all patients).

With regard to the 5 dimensions on the EuroQuol-5, severe problems with “mobility” were not reported by any patient: 26 (56%) patients reported moderate problems with mobility compared to 20 (44%) that reported no problems (Figure 2). Only 2 patients (4%) reported severe problems in the “self-care” domain; a majority of patients, 31 (67%), reported no problems with self-care. Eleven patients (24%) reported severe problems with “usual activities,” while 23 (50%) reported moderate problems. When asked about “pain or discomfort,” no patients reported severe problems, but the majority 38 (83%) did indicate having moderate problems with pain or discomfort. On the question of “anxiety/depression,” only one patient (2%) reported severe problems with anxiety or depression, while the majority of patients, 35 (76%), reported no problems.  Table 3 summarizes the EQ-5 data collected from each group.

There were two Emergency Room visits recorded, one of which was readmitted to the hospital for incision and drainage of a perineal hematoma. No unscheduled clinic visits were identified.

In light of increasing emphasis on patient reported outcome measures (PROMs), a concerted effort has been made to have a questionnaire specific to urethral stricture disease. This has culminated in Jackson et al. developing the validated urethral stricture PROM, part of which assesses health-related quality of life [11]. Prior to that, various tools developed for other disease states were utilized for the urethral stricture patient [12]. We utilized the EuroQuol-5 validated questionnaire as it seeks to identify general health-related difficulties these patients may face, particularly in the context of an elective procedure (urethroplasty) intended to improve quality of life. Most patients, 82.6%, did report moderate problems with pain and discomfort. However, despite the added morbidity of buccal harvest in most of the patients, none reported severe pain within 24 hours after discharge. In this population the donor site was left open; however, there are several studies with contradicting conclusions on effect of donor site closure on postoperative pain [13–16].

In this cohort, the majority of patients reported moderate and severe problems in performing usual activities (74%). This was expected as the patients were sent home with an indwelling catheter for 3 weeks and strict instructions to avoid strenuous physical activity and abstain from any sexual activity. Given the varying types of urethroplasty performed in this small population it is difficult to ascertain whether the type of procedure correlates with the increased perception of pain postoperatively. One patient reported severe problems with anxiety or depression, which was unexpected considering that the procedure was performed with a goal of improving the patient's quality of life. This finding highlighted an important limitation of this study, a lack of preoperative data on patients' baseline health-related quality of life. We have since changed our practice and administer all PROM questionnaires pre- and postoperatively.

To our knowledge, there are no published studies on patient-reported perception of appropriateness of timing of discharge after anterior urethroplasty. The only studies on short-stay or outpatient urethroplasty published by Lewis et al. and MacDonald et al. have concentrated on clinical outcomes [5, 6].

Results of anterior urethroplasty performed in the outpatient setting were first described in 2002 by Lewis et al. [5]. The authors described a cohort of patients who underwent bulbar urethroplasty and were then discharged home within 23 hours of surgery. In 2006, MacDonald et al. published outcomes of the “same day urethroplasty,” which he defined as being discharged home within 4 hours after surgery [6]. In both series the outcomes of the surgery were excellent but the cohorts were small.

In detail, the first study described 78 bulbar urethroplasties of which 54 (69%) were performed on a short-stay basis (patients discharged <24 hours after surgery) [5]. Overall success in the short-stay cohort was 93% compared with 88% of the admitted inpatient cohort. The authors noted that the short-stay status depended on the type of urethroplasty (90% after EPA, 64% after penile skin flaps, and 45% after buccal mucosal grafts), younger patient's age (36 versus 46 years), and shorter stricture length (3.1 versus 6.6 cm.). The study did not comment on readmissions, ER visits, or unscheduled clinic visits.

In the second study, MacDonald et al. retrospectively describes 54 patients after anterior urethroplasty performed over 4 consecutive years [6]. Over the study period, the rate of the outpatient (same day) urethroplasty increased from 27% to 85%. In this study the outpatient and the admitted inpatient cohorts had similar stricture length, but the outpatient cohort was slightly younger age (42 versus 49 years of age). Over the 27 months of follow-up the success rate was similar in both groups (94% versus 97% in the inpatient group) as were the long-term complications (19% versus 18%, resp.). The authors reported that no readmissions or emergency room visits occurred in this study.

For both studies, overall clinical outcomes were similar between the outpatient or short-stay group and admitted patients. These two studies represent the only studies published on “minimal-impact urethroplasty” and further evaluation of outpatient urethroplasty, as far as patient reported outcome measures have been lacking.

In our series, the majority of patients were comfortable with the timing of discharge in both the outpatient and short-stay cohorts. Given the relative small size of the outpatient cohort, we did not attempt further statistical comparison of the two groups. Additionally, the decision to leave or stay was made by the patient and as might be expected the majority of patients later agreed with their own choices. We surmised that the few “too soon” responses represented a later regret of their original decision. Overall, majority of patients were satisfied with leaving the hospital within 23 hours after urethral reconstruction, even for long or panurethral strictures requiring extensive dissection and bilateral BMG harvest. This data is reassuring as it shows that majority of patients did not feel rushed out of the hospital. This study can serve for a future counseling of patients considering a short-stay urethroplasty showing it as a reasonable option from patients' perspective.

With prompt postoperative discharge, there is a concern about increased readmission rates; this failed to materialize in this series [17]. In our cohort there were two ER visits, one of which was related to patient's concern of scrotal bruising and another for perineal hematoma. The latter resulted in the only readmission to the hospital and subsequent incision and drainage. There were no unscheduled visits to the outpatient clinic in this group showing that early discharge from the hospital did not shift the burden of care from the inpatient to outpatient setting.

Some limitations of the study include its retrospective nature and the nonrandomization of the two groups, which led to an uneven distribution of the outpatient versus short-stay groups. This limited the ability to perform a multivariate or comparative analysis for each group. No preoperative EuroQuol-5 questionnaires were administered making it difficult to identify patients with preexisting problems in any of the 5 dimensions. This study is limited by the assumption that every patient was in sufficiently good health prior to surgery. However, even with this assumption, the majority of patients did not report severe changes in the health-related quality of life shortly after urethroplasty.

## 4. Conclusion

Early return home after urethroplasty seems to be well tolerated by patients as reported on their health-related quality of life questionnaire. When using EQ-5 as a quality of life indicator in the early postoperative period, the patient's QOL was only minimally affected, except when otherwise expected in domains of “pain” and “usual activities.” Most patients are satisfied with timing of their discharge from the hospital after a short-stay or outpatient urethroplasty. Early discharge did not result in numerous catastrophes leading to ER visits, readmissions, or unscheduled office visits.

## Figures and Tables

**Table 1:** Patient characteristics.

	Mean (Std. Dev.)	Range
Age (years)	51.6 (±15.65)	21–78
BMI (kg/m^2^)	30.6 (±6.2)	18.6–44.7
Stricture length (mm)	60 (±51)	5–200

Stricture etiology	Number	%

Idiopathic	24	50
Trauma	9	19
Iatrogenic	9	19
Infectious	2	4
Radiation	2	4
Lichen sclerosis	2	4

Stricture location	Number	%

Bulbar	20	42
Bulbomembranous	12	25
Panurethral	9	18
Pendulous	5	10.4
Fossa navicularis	2	4

**Table 2:** Type of urethroplasty.

Repair type	Number (%)
One sided dissection, dorsal onlay (Kulkarni)	13 (27%)
Excision and primary anastomosis (EPA)	11 (23%)
Augmented anastomotic urethroplasty (AAU)	10 (21%)
Dorsal onlay	9 (19%)
Ventral onlay	4 (8%)
Others	1 (2%)

**Table 3:** EQ-5 patient responses by group.

EQ-5D dimension	All responders (%)	Outpatient (%)	Short-stay (%)
Mobility			
1 = no problem	20 (44%)	3 (43%)	17 (56%)
2 = moderate	26 (56%)	4 (57%)	22 (44%)
3 = severe	0	0	0
Self-care			
1 = no problem	31 (68%)	6 (86%)	25 (64%)
2 = moderate	13 (28%)	1 (14%)	12 (31%)
3 = severe	2 (4%)	0	2 (5%)
Usual activity			
1 = no problem	12 (26%)	3 (43%)	9 (23%)
2 = moderate	23 (50%)	3 (43%)	20 (51%)
3 = severe	11 (24%)	1 (14%)	10 (26%)
Pain/discomfort			
1 = no problem	8 (17%)	1 (14%)	7 (18%)
2 = moderate	38 (83%)	6 (86%)	32 (82%)
3 = severe	0	0	0
Anxiety/depression			
1 = no problem	35 (76%)	7 (100%)	28 (72%)
2 = moderate	10 (22%)	0	10 (26%)
3 = severe	1 (2%)	0	1 (2%)
